# Supplementary material for: Involving Parents in Promoting Healthy Energy Balance-Related Behaviors in Preschoolers: A Mixed Methods Impact and Process Evaluation of SuperFIT
Source: Nutrients. 2021 May 11;13(5):1605. doi: 10.3390/nu13051605 (PMC8150277; doi:10.3390/nu13051605)
Supplement: Supplementary file 1 [file nutrients-13-01605-s001.zip › nutrients-1169324-supplementary.pdf]

Table S1. Self-reported physical home environment of parents participating in the FC.

PA equipment	Max score	Baseline (n=28)		Follow-up 1 7-10 months (n=22)		Follow-up 2 13-16 months (n=25)	
		Yes (%)	Mean score	Yes (%)	Mean score	Yes (%)	Mean score
**Access to a garden**		92.9		86.4		92.0	
Natural elements (e.g. tree)	9		3.4		4.7		3.6
**Equipment total**							
Inside	16	100.0	4.8	100.0	5.1	100.0	4.5
Outside	13	96.4	6.5	100.0	6.7	100.0	7.5
**Fixed play equipment** [1]							
Inside	5	16.7	0.1	21.1	0.4	15.8	0.2
Outside	5	89.3	1.7	89.5	2.0	87.0	2.5
**Portable equipment** [2]							
Inside	6	89.3	1.6	100.0	1.9	86.4	1.6
Outside	5	96.4	3.8	100.0	3.8	100.0	3.9
**Sedentary equipment** [3]	3	100.0	1.9	100.0	1.8	100.0	1.9

[1] Fixed equipment included: markings on the ground, sandbox, climbing frame, slide, and swing. [2] Portable equipment included: balls or other materials to throw or roll, soccer goal or basketball ring, skipping rope or other materials to jump with, walkable car or other materials to drive, sand or water materials, and other portable materials. [3] Sedentary equipment included: televisions and computer devices.
